# Web-Based Intervention to Teach Developmentally Supportive Care to Parents of Preterm Infants: Feasibility and Acceptability Study

**DOI:** 10.2196/resprot.8289

**Published:** 2017-11-30

**Authors:** Thuy Mai Luu, Li Feng Xie, Perrine Peckre, Sylvana Cote, Thierry Karsenti, Claire-Dominique Walker, Julie Gosselin

**Affiliations:** ^1^ Research Center Centre Hospitalier Universitaire Sainte-Justine Montreal, QC Canada; ^2^ Department of Pediatrics University of Montreal Montreal, QC Canada; ^3^ Department of Social and Preventive Medicine University of Montreal Montreal, QC Canada; ^4^ Faculty of Teaching and Education Sciences University of Montreal Montreal, QC Canada; ^5^ Douglas Mental Health University Institute Department of Psychiatry McGill University Montreal, QC Canada; ^6^ School of Rehabilitation University of Montreal Montreal, QC Canada

**Keywords:** early intervention, developmental intervention, preterm infants, neurodevelopmental outcomes, Web-based intervention, Internet

## Abstract

**Background:**

Preterm birth affects 8% to 11% of the population and conveys a significant risk of developmental delays. Intervention programs that support child development have been shown to have a positive impact on early motor and cognitive development and on parental well-being. However, these programs are often difficult to implement in a real-life setting due to lack of resources. Hence, our multidisciplinary team developed Mieux Agir au Quotidien (MAQ) to teach developmentally supportive care to parents of preterm infants with the goal of improving child development and parental outcomes. Our intervention included 3 in-person workshops that occurred prior to hospital discharge and a Web-based platform with written and videotaped materials that addressed 5 main themes: (1) infant behavioral cues, (2) flexion positioning; (3) oral feeding support, (4) parent-infant interactions, and (5) anticipation of developmental milestones.

**Objective:**

This study aimed to test the feasibility and acceptability of the intervention by parents of preterm infants and assess clinical benefits on child neurodevelopment and parental outcomes during the first year of life.

**Methods:**

A total of 107 infants born at <30 weeks and admitted to Sainte-Justine Hospital neonatal intensive care unit and their parents were enrolled in a nonrandomized controlled before-and-after interventional study (intervention n=55, comparison n=52). Acceptability of the program was assessed with a user satisfaction questionnaire. When the infants were at 4 months’ corrected age, all parents completed questionnaires on infant temperament, parenting stress, sense of competence, and parenting satisfaction. At 12 months’ corrected age, neurodevelopmental testing was performed on infants using the Alberta Infant Motor Scale and the Bayley Scales of Infant and Toddler Development, Third Edition. Comparisons between the 2 groups were done using independent *t* tests, Wilcoxon rank-sum tests, and Fisher exact tests.

**Results:**

The majority of parents (43/45) were satisfied with the intervention program and all would recommend MAQ to others. MAQ met their need for evidence-based information that proved useful to support their child development. No difference in parental or child neurodevelopmental outcomes was detected in this pilot study for most outcomes except for higher median scores for parental coercive behaviors in the intervention group, although proportions scoring in the coercive range did not differ.

**Conclusions:**

Acceptability of the program was high among parents thus supporting the relevance of such intervention. A larger study using a randomized controlled trial design is needed to better document impact on parent and children and investigate how Web-based technologies can efficiently complement individualized intervention to alleviate the burden on health care resources.

## Introduction

### Preterm Birth: Risk Factor for Neurodevelopmental Impairment

Medical progress has led to an increase in preterm birth (occurring <37 weeks of gestation) along with improvement in survival of the sickest and most immature babies. However, rates of neurodevelopmental impairment remain high, with half of preterm children born <32 weeks of gestation exhibiting a spectrum of morbidities affecting motor, cognitive, language, socioemotional, and behavioral development [1].

Although prematurity-related medical complications account for some of the variability in neurodevelopmental outcomes [2], the preterm child’s physical and social environment also significantly influences brain development [3]. After the preterm infant is born, exposure to various noxious sensory stimulations (loud noise, bright light, pain, invasive oral stimulation, prolonged restrictive positioning) occurs in the neonatal intensive care unit (NICU). The infant’s immature neuronal circuitry is not ready to process these overwhelming physical stressors, which may affect normal brain development [3]. In addition, the quality of parent-infant interaction is challenged after preterm birth due to unanticipated separation [4]. Together, these environmental and social factors may hinder healthy brain development in preterm infants.

### Developmental Intervention to Improve Neurodevelopmental Outcome

Developmental interventions aim to support infant development through educational strategies or techniques that promote the emergence of new skills and competences while preventing delays and disabilities [5]. These interventions take advantage of infant brain plasticity which is thought to be maximal between 28 and 32 weeks postconception to 2 years of age [6]. Developmental interventions in preterm infants commonly address adaptation of the physical environment to improve sensory inputs, enhancement of parent-infant interactions, and infant stimulation through, for example, neurodevelopmental therapy [7]. Existing developmental interventions for preterm infants are numerous and all differ in terms of timing, intensity, setting, structure, and resources.

Meta-analyses suggest that developmental interventions in preterm infants enhance cognitive and motor outcomes during the early years [7-9], although sustainability of these improvements has only been shown in one study [10]. Benefits on maternal anxiety, depressive symptoms, and self-efficacy have also been documented [11]. Moreover, individual intervention programs have shown positive effects on parent-infant interactions [12-14] and child behavior [14-18]. Developmental interventions with the most promising results share the following key components: parent-mediated intervention, parenting education, adaptation of the environment to reduce stressful stimuli, enhancement of parent-infant interactions, and psychosocial support [9,11,19].

Despite the benefits of developmental interventions, human and financial resources required to implement such endeavors often hinder implementation in a real-life setting [20]. In recent years, Web-based technologies have emerged as an alternative to deliver self-management or parenting interventions with proven effectiveness in chronic health conditions such as asthma and traumatic brain injury [21,22]. Web-based interventions have a high potential for outreach at lower costs. To our knowledge, there are no such programs for parents of preterm infants.

### Mieux Agir au Quotidien: Multimodal Approach Using Web-Based Technologies to Deliver a Developmental Intervention

#### Overview

*Mieux Agir au Quotidien* (MAQ) is designed to empower parents to create an enriching physical and social environment for their infant using developmentally sound practices. It targets parents of preterm infants at risk of developmental delays. To improve efficiency of program delivery with a minimum of resources, MAQ uses a multimodal approach that combines educational support from a developmental intervener along with a Web-based platform that serves to provide and consolidate acquired knowledge [23]. The program is divided into 5 task-oriented teaching-learning modules readily available at all time in the NICU and at home through the Internet (Textbox 1). The 5 modules address (1) interpretation of behavioral cues and environmental adaptation, (2) adapted flexion positioning, (3) oral feeding support, (4) enhancement of parent-infant interactions through play, and (5) anticipatory guidance of preterm infant development and stimulation activities. All modules are structured similarly and explain the relevance of each theme to child development, provide some theoretical background, and teach developmental and behavioral activities that can be easily replicated in everyday life to consolidate knowledge acquisition. The program can be initiated when the infant reaches 32 weeks of postmenstrual age (Table 1). While the baby is still in the NICU, parents are introduced to developmentally supportive care through 3 hour-long in-person workshops that cover interpretation of behavioral cues, adapted flexion positioning, and parent-infant interactions. These workshops are delivered by the developmental intervener and use the Web-based platform as a visual support. Parents are encouraged at each session to go through the first 4 Web-based modules, which contain both written and visual contents illustrating hands-on activities that can be readily applied in the NICU (pictures, videos). Moreover, posters illustrating key concepts (behavioral cues and flexion positioning) are placed in the NICU as an additional reminder.

**Overview of the Web-based teaching modules of Mieux Agir au Quotidien.**

Reading my child’s behavior:

- Understanding sensory development
- Recognizing the different sleep-arousal states and the importance of sleep protection
- Interpreting infant behavioral cues (stress and self-regulatory behaviors)

Bringing the infant to an organized and stable state (control of environmental stimuli, skin-to-skin holding, nonnutritive sucking)

Positioning my child in the right position:

- Understanding muscle tone and motor development in preterm infants
- Recognizing abnormal postures that can lead to muscle and bone deformities

Promoting adapted flexion positioning and optimal posture during sleep, play time, and daily activities

Supporting oral feeding:

- Understanding developmental milestones related to oral feeding
- Understanding feeding challenges in preterm infants

Facilitating oral feeding (nonnutritive sucking, flexion positioning, pacing)

Playing and interacting with my child:

- Understanding attachment development
- Understanding how an infant temperament can influence parent-infant relationships
- Promoting play time

Creating an environment that will facilitate interactions and fun play

Foreseeing my child’s developmental milestones:

- Understanding child developmental milestones and diversity of developmental trajectories
- Recognizing developmental red flags

Promoting neurodevelopmental stimulation

**Table 1:** Timing of intervention.

Schedule	In the neonatal intensive care	Home
Timing	Starting at 32 weeks of gestational age.	From term-equivalent age to 1 year of age.
Number of sessions	Three in-person workshops: (1) infant behavioral cues, (2) flexion positioning, (3) parent-infant interactions. Four Web-based modules: (1) infant behavioral cues, (2) flexion positioning, (3) oral feeding support, (4) parent-infant interactions.	Consolidation of first 4 Web-based modules. Fifth Web-based module on anticipation of developmental milestones.

After hospital discharge, parents can continue to go through all 5 Web-based modules containing educational material with new stimulation activities that can be done at home and are applicable throughout the first year of age. At all times, they can reach the developmental intervener through email.

MAQ is guided by 2 principles: early developmental care and enhancement of parent-infant interactions.

#### Early Developmental Care

Early developmental care is the cornerstone of many developmental interventions. Early developmental care aims to reduce stress and optimize developmentally appropriate stimulation [24,25]. Different strategies are employed to promote infant well-being and include control of external stimuli (adjusting ambient light, reducing noise), clustering of care and sleep protection, appropriate flexion positioning and handling (such as skin-to-skin holding), oral feeding support (including nonnutritive sucking), and parent involvement. By tailoring care to the infant’s needs and behavioral state, his or her energy expenditure is kept at a minimum and can be redirected to optimize growth and development [24]. MAQ provides parents with specific modules that teach these evidence-based techniques of early developmental care.

#### Enhancement of Parent-Infant Interactions

Child developmental outcomes are shaped by the continuous dynamic interactions of the child and the immediate social experience provided by the family [26]. This transactional model of development highlights how the child influences his or her environment (including caregivers), which in turn affects the child, and so on. Preterm birth can significantly alter parent-infant interactions. On one hand, preterm infants often display poorly regulated behaviors that are exacerbated by noxious stimuli (bright light, pain, noise, etc). This will interfere with social interactions, notably with parents [27]. On the other hand, parents of preterm children have been shown to be at increased risk of psychological distress and report higher levels of parenting stress, especially when faced with an infant with difficult temperament [28,29]. This can impede nurturing behaviors and negatively shape the infant’s experience. MAQ teaches parents about interpretation of infant behavioral cues (stress/avoidance vs approach/self-regulatory behaviors) and how to sensitively respond to them during key moments such as sleep and feeding. In addition, a module is dedicated to techniques during play time to enhance shared positive parent-child experiences and promote bonding.

MAQ is novel for its key combination of parents’ active involvement and developmentally supportive care using an easily accessible Web-based interface. However, before we launched MAQ in practice, we conducted a study whose objectives were to examine feasibility and acceptability of our pilot educational intervention and assess its clinical benefits on infant development and parental well-being. This study was granted approval by the Sainte-Justine Hospital Research and Ethics Committee.

## Methods

### Study Design and Population

This was a nonrandomized before-and-after intervention feasibility study that compared a historical group to an intervention group that received the program. All families with preterm infants born <30 weeks of complete gestation from 2010 to 2013, admitted at Sainte-Justine Hospital NICU, Montreal, Canada, and surviving to 32 weeks’ postmenstrual age were eligible for participation. Exclusion criteria were: (1) chromosomal anomalies and major congenital malformation, as the pathophysiology for neurodevelopmental impairment is different, (2) documented parental history of recent illicit drug use, alcoholism, severe mental illness, intellectual disability, or domestic violence, given the added adverse effects on brain development and for adherence and consent issues, (3) foster care placement, for consent issues, (4) no family member speaking French (English translation had not taken place at that time), or (5) patients doomed to die within a few days, as estimated by the attending physician.

### Procedure

The historical nonexposed comparison group comprised 52 infants born from 2010 to 2011 who were recruited after discharge from the hospital. Before implementation of MAQ in the NICU at Sainte-Justine Hospital, standardized protocols of developmentally supportive care were scarce and included nonmandatory workshops on skin-to-skin holding. After neonatal discharge, families were seen for routine neurodevelopmental assessment at the neonatal follow-up clinic where referrals for medical or early intervention services could be made depending on needs.

The intervention group consisted of 55 infants born from 2012 to 2013, enrolled when they reached 32 weeks of gestational age, prior to hospital discharge. Upon recruitment, parents received a unique identifier that allowed them to access the Web-based platform to start the educational intervention program. A computer was made available to parents at all time in the NICU. We also printed the written material available on the Web as requested by some parents. In-person workshops were then scheduled with the developmental intervener who was a board-certified occupational therapist trained in developmental care. After the infant was discharged, the developmental intervener maintained contact through phone or email to encourage parents to access the Web-based modules (around 4, 8, and 12 months’ corrected age [CA]). Of note, all families had home access to a computer and an Internet connection. Families were also seen when possible at the neonatal follow-up clinic as part of standard of care (1-2, 4, and 8-9 months’ CA).

For this study, participants were seen at 4 and 12 months’ CA for outcome assessment at Sainte-Justine Hospital.

### Outcome Measures

#### Measures of Feasibility and Acceptability

For families enrolled in the MAQ intervention program, compliance and use of the different modules were monitored by attendance records at workshops and self-reported access to the Web-based platform. Parental satisfaction regarding the contents and structure of the Web-based component of the educational program was appraised using an adapted version of the User Satisfaction Questionnaire [30], in which parents were asked to rate on a 4-item scale their opinion on 15 statements. An overall mean score of 45 indicates user satisfaction with the Web-based application. Parental concerns and beliefs regarding the program were also addressed through open-ended questions, specifically (1) what they generally thought about the workshops and Web-based platform, (2) what were perceived barriers and facilitators to access the Web-based platform, (3) what were their suggestions for improvements.

#### Measures of Parental Outcomes

When the child was 4 months’ CA, parents were asked to complete the Parental Cognitions and Conduct Toward the Infant Scale (PACOTIS) [31] that measures parental perceptions and behavioral tendencies toward a recently born infant. There are 4 subscales: parental self-efficacy and perceived parental impact center on parents’ beliefs about their role as a parent, whereas parental coercive behaviors and parental overprotection reflect behavioral tendencies toward the infant. A higher score indicates greater endorsement of a given parenting dimension.

Parents also filled the Parenting Stress Index–Short Form (PSI) [32], a well standardized and validated questionnaire that yields a total stress score from 3 scales: parental distress, parent-child dysfunctional interaction, and difficult child. The PSI identifies dysfunctional parenting and predicts the potential for parental behavior problems and child adjustment difficulties.

#### Measures of Infant Neurodevelopmental Outcome

Child temperament was assessed at 4 months’ CA using the Bates’ Infant Characteristic Questionnaire [33], a self-administered questionnaire in which parents indicate the level of perceived difficulty of their child in dealing with specifically described behaviors.

At 12 months’ CA, cognitive, language, and motor development was assessed by a trained occupational therapist using the Bayley Scales of Infant and Toddler Development (BSITD), 3rd edition [34], a widely used norm-referenced and standardized instrument. The BSITD yields 3 scales—cognitive, language, and motor—with a mean of 100 and a standard deviation of 15. Finally, the Alberta Infant Motor Scales [35], an observational and standardized measure of motor development, was performed by physical therapists.

### Statistical Analyses

As this was a pilot study to primarily test the feasibility and acceptability of our educational intervention program, with 50 infants per arm, the power to detect a difference in developmental scores of 0.3 to 0.5 standard deviations between the intervention and comparison groups at a 2-sided alpha level of .05 was between 38% and 70%.

Qualitative comments from questionnaires were analyzed by 2 independent reviewers (TML, PP) using thematic analysis and coding. To summarize the study population characteristics, descriptive statistical analysis was performed on baseline perinatal and sociodemographic factors using means and standard deviations, medians and range, and proportions. Comparisons were made using independent *t* tests (normally distributed continuous variables), Wilcoxon sum-rank tests (continuous variables not normally distributed), and chi-square or Fisher exact tests (categorical variables). Statistical analyses were performed with SPSS 24.0 (IBM Corp).

## Results

### Participant Characteristics

**Figure 1:**
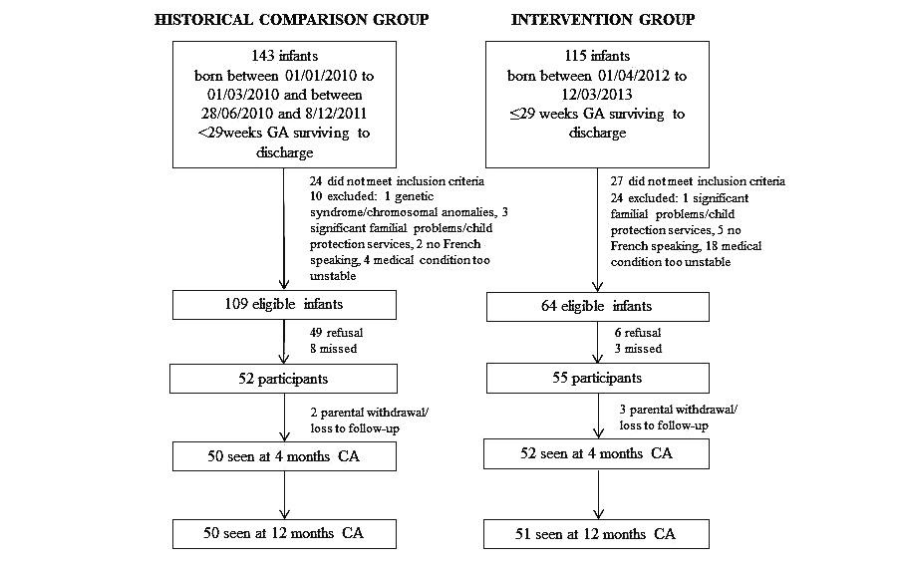
Flow diagram of study population.

Overall, 107 infants and 96 mothers (45 in the comparison group, 51 in the intervention group) were recruited to the study (see study flow diagram in Figure 1). Table 2 describes sociodemographic and neonatal characteristics. Infants in the intervention group, compared to the historical cohort, were of higher gestational age and birth weight and were less likely to have severe retinopathy of prematurity. Therefore, their baseline risk of adverse neurodevelopmental outcomes was lower [36].

Participation in the intervention program was high with 44/51 mothers attending workshops and 45/51 going through the Web-based educational modules. In total, 48/51 parents (94%) participated in the program.

**Table 2:** Sociodemographic and neonatal characteristics of the study population.

Characteristics				Comparison N=45 parents N=52 infants	Intervention N=51 parents N=55 infants
**Parent**					
	**Maternal education, n (%)**				
		High school and less	5 (11)		12 (24)
		Some college	20 (44)		15 (29)
		University	20 (45)		24(47)
	Single parent household, n (%)			4 (9)	4 (8)
	White, n (%)			36 (80)	35 (69)
	**Paternal education, n (%)**				
		High school and less	11 (27)		12 (26)
		Some college	14 (33)		14 (30)
		University	16 (38)		20 (44)
**Infant**					
	Gestational age, weeks, mean (SD^a^)			25.6 (1.5)	27.5 (1.4)
	Birth weight, g, mean (SD)			917 (207)	1088 (233)
	Male, n (%)			28 (54)	34 (62)
	Multiple gestation, n (%)			12 (23)	10 (18)
	Small for gestational age, n (%)			6 (12)	4 (7)
	Bronchopulmonary dysplasia, n (%)			33 (63)	28 (51)
	Sepsis, n (%)			20 (38)	21 (38)
	Surgical necrotizing enterocolitis, n (%)			2 (4)	0
	Severe retinopathy of prematurity, n (%)			10 (19)	2 (4)
	Severe brain lesions on ultrasound, n (%)			2 (4)	6 (11)
	Duration of neonatal hospitalization, days, mean (SD)			102 (41)	89 (31)

^a^SD: standard deviation.

### Overall Satisfaction With the Intervention Program

The User Satisfaction Questionnaire was completed by 45 parents in the intervention group (Multimedia Appendix 1). Satisfaction with the Web-based component was good with an overall mean score of 51 (minimum 44 and maximum 60). The majority of parents (43/45) rated the Web application above the acceptable range (≥45), with 2 parents giving a score of 44. All parents agreed (24/45, 53%) or strongly agreed (21/45, 47%) with the statement on overall satisfaction with the Web-based tutorials, and all would recommend them to other parents of preterm infants. A small number of parents reported that the amounts of examples and illustrations were insufficient (3/45) and disagreed with the statement that “technology was as effective as traditional teaching methods in helping them learn the material” (5/45). Narrative comments were provided by 10 mothers about the entire intervention program. Parents invoked 3 main themes: (1) information (ie, intervention responded to their need for information), (2) complementarity (ie, using different teaching approaches [workshop, Web-based modules, posters] was important), (3) concrete examples (ie, more day-to-day examples of problems and solutions should be provided). With regard to the latter, parents notably enjoyed the module on “Positioning my child” as it was problem-based and practical, whereas the module on “Supporting oral feeding” was deemed to be too theoretical. In relation to information, it was considered important that teaching material be presented in a nonjudgmental and nondirective way (eg, “Don’t say that we should, but rather that we could”).

### Parental Outcomes

In total, 43 parents of 48 infants in the comparison group and 42 parents of 45 infants in the intervention group responded to questionnaires when their infants reached 4 months’ CA (Table 3). Parents in the intervention group had a higher median score on the coercive behavior scale, but there was no difference in proportion scoring in the coercive range. Moreover, no difference was detected on the other scales of the PACOTIS or on the Parenting Stress Index.

**Table 3:** Parental outcomes at infant’s 4 months corrected age.

Outcome			Comparison (N=43)	Intervention (N=42)	*P* value
**PACOTIS^a^**					
	**Self-efficacy**				
		Parental self-efficacy, median (IQR^b^)	8.8 (7.8-9.3)	8.8 (8.2-9.2)	.57
		Low self-efficacy, n (%)	11 (26)	8 (19)	.47
	**Impact**				
		Perceived parental impact, median (IQR)	9.0 (7.2-10.0)	8.1 (7.0-9.8)	.59
		Low parental impact, n (%)	6 (14)	7 (17)	.73
	**Coercion**				
		Parental coercive behaviors, median (IQR)	0.4 (0-1.4)	1.3 (0.3-2.1)	.04
		Coercive parenting, n (%)	6 (14)	10 (24)	.25
	**Overprotection**				
		Parental overprotection, median (IQR)	5.2 (3.0-6.4)	4.3 (3.0-6.8)	.33
		Overprotection, n (%)	9 (21)	8 (19)	.83
**Parenting Stress Index**					
	Total stress score, median (IQR)		63 (54-70)	62 (50-82)	.82
	Parental distress, median (IQR)		24 (19-28)	26 (19-31)	.82
	Parent-child dysfunctional interaction, median (IQR)		18 (14-21)	16 (14-21)	.39
	Difficult child, median (IQR)		22 (16-25)	20 (16-26)	.82

^a^PACOTIS: Parental Cognitions and Conduct Toward the Infant Scale.

^b^IQR: interquartile range.

**Table 4:** Infant neurodevelopmental outcomes at 12 months of corrected age.

Outcome		Comparison (N=50)	Intervention (N=51)	*P* value
**Alberta Infant Motor Scales**				
	Total score, median (min-max)	47 (33-56)	49 (6-58)	.53
	Score <10th percentile, n (%)	22/49 (45)	21/51 (41)	.71
**BSITD^a^**				
	Cognition score, mean (SD^b^)	96 (9)	97 (13)	.77
	Motor composite score, mean (SD)	92 (11)	89 (13)	.22
	Gross motor scale, mean (SD)	9 (3)	8 (3)	.78
	Fine motor scale, mean (SD)	9 (2)	9 (2)	.18
	Language composite score, mean (SD)	90 (12)	96 (13)	.74
	Expressive language scale, mean (SD)	8 (2)	9 (3)	.27
	Receptive language scale, median (min-max)	9 (4-11)	9 (1-15)	.16
	Cognition score <85, n (%)	3/50 (6)	6/48 (13)	.20
	Motor composite score <85, n (%)	10/49 (20)	10/48 (22)	.76
	Language composite score <85, n (%)	13/50 (26)	7/48 (15)	.26

^a^BSITD: Bayley Scales of Infant and Toddler Development.

^a^SD: standard deviation.

### Infant Neurodevelopmental Outcomes

At 4 months’ CA, there was no difference in mean scores on parental perception of infant temperament (comparison: 23 [SD 6], intervention: 23 [SD 7]). In the comparison group, 38/46 (83%) infants were described as fussy versus 34/47 (73%) infants in the intervention group. We did not find any difference on motor, cognitive, and language development between the 2 groups at 12 months’ CA (Table 4).

## Discussion

### Principal Findings

This study aimed to assess feasibility and acceptability of this new multimodal educational program of developmentally supportive care for parents of preterm infants. This program is unique for its Web-based platform, and is, to our knowledge, one of the first to offer such educational material. The majority of parents participated into the educational program, and the majority were also satisfied with the clinical contents and format, thus supporting feasibility and acceptability of such intervention. However, parents in the intervention group reported more coercive tendencies toward their infant when compared to the historical cohort, although proportion scoring in the abnormal range did not differ. Analysis of preliminary results did not reveal any difference on parenting stress or infant neurodevelopmental outcomes at 12 months’ CA.

Existing developmental intervention programs for infants born preterm require substantial human resources, which can represent a challenge with regard to implementation. For example, promising programs such as the Mother-Infant Transaction Program and modified versions [14,37,38], the Infant Behavioral Assessment and Intervention Program [39] and the Victorian Infant Brain Studies Plus [40], to name a few, include from 7 to 10 individual sessions, some of which are conducted during home visits. Although ideal, this may not be feasible due to shortage of qualified personnel or geographical reasons, when long distances need to be covered for home visits. We attempted to bridge this gap by combining in-hospital face-to-face sessions with a Web-based tutorial for parents of preterm infants. Indeed, all parents in our study endorsed the Web-based component of the program and would recommend it to others as it responded to a need for scientifically sound information about developmentally supportive care.

### Limitations

However, some weaknesses were identified. Although 44/51 parents attended workshops, great efforts from our developmental intervener were required to plan these sessions. Indeed, flexibility in the schedule was crucial to accommodate parents’ visiting hours. In addition, workshops often ended as individual bedside sessions as parents were understandably reluctant to leave their preterm infant. Therefore, in a real-life setting, significant resources and time would still need to be allocated to ensure in-person delivery of program contents with compliance likely to be well below the observed 86% in our study.

Another limitation was that most parents accessed the Web-based platform during their neonatal stay when they had more spare time and not at home as they became busier and started forgetting about the available resource. To improve uptake of the program at home, parents need to become more familiar with the Web-based component prior to discharge, which can only occur with increasing use. Barriers to use included easy access to a computer and Internet connection at our hospital at the time of the study (2012-2013). Although a computer was available, it could not be used at bedside. Nowadays, wireless Internet access is becoming more widespread in hospitals in industrialized countries. The website has also been made compatible with mobile phones and tablets. Furthermore, we addressed parents’ feedback to improve the contents and formats of the Web-based component of MAQ to make it more appealing. All written material has been re-edited so messages would be clearer and less directive. More tables, figures, and videos have been created to facilitate learning and understanding of the material (Multimedia Appendices 2 and 3). Translation to English has been completed to broaden the audience. It has also been suggested that personalized reminders be sent by emails or text messages with links directing parents to age-appropriate learning material. This is in our long-term goals to improve MAQ.

Preliminary results on parent and infant outcomes must be interpreted with caution, especially the finding of higher scores for parental coercive behavior in the intervention group. However, proportions with coercive tendencies did not differ between groups. Our exploratory study used a nonrandomized design which made it vulnerable to selection bias. Indeed, infant and parent characteristics were not balanced across groups. Infants in the historical comparison cohort were of lower gestational age and birth weight and more likely to have severe retinopathy of prematurity, whereas their parents were more likely to have completed higher educational levels. The small sample size limited our ability to adjust for all these factors in our analyses. Second, we did not examine parents’ behavioral characteristics at study entry, and it is possible that parents in the intervention group differed in terms of their baseline personal traits, which could have influenced their answers to the parental questionnaires. This can only be addressed with a pilot randomized controlled trial. We did not find any difference between groups in motor development as measured by the Alberta Infant Motor Scales nor did we detect cognitive, language, or motor improvements on the BSITD following the intervention. As this was a feasibility and acceptability study, it was underpowered to find small or even moderate differences between groups. Moreover, other studies suggest that the greatest benefits may be on behavioral outcomes [17,18]. A future study would therefore need to assess child behavior at later ages. In addition, it would also require assessment of improvement in parental knowledge at different time points as a first step in the application of the educational material. Finally, current results are only applicable to the subset of very preterm infants without other medical risk factors such as chromosomal abnormalities or social risk factors including parental mental health issues and foster care placement.

### Conclusions

We have shown the feasibility and acceptability of using a multimodal approach including Web-based applications to deliver an educational intervention on developmentally sound practices for parents of preterm infants. Parental input regarding weaknesses of the Web-based component of the program has been since carefully considered to significantly improve contents and format. With increasing use of the Internet as a source of knowledge, the *Mieux Agir au Quotidien* website is highly relevant to respond to parents’ needs for reliable and evidence-based information. Results from this preliminary study can now serve to inform a future pilot randomized controlled trial.

## Appendix

Multimedia Appendix 1 User Satisfaction Questionnaire (n=45).

Multimedia Appendix 2 Examples of material on the Web-based component of Mieux Agir au Quotidien.

Multimedia Appendix 3 Examples of material on the Web-based component of Mieux Agir au Quotidien.

Multimedia Appendix 4 Peer review and funding report from SickKids Foundation and the Institute of Human Development, Child and Youth Health.

## Abbreviations

BSITD: Bayley Scales of Infant and Toddler Development
CA: corrected age
MAQ: Mieux Agir au Quotidien
NICU: neonatal intensive care unit
PACOTIS: Parental Cognitions and Conduct Toward the Infant Scale
PSI: Parenting Stress Index
