# Supplementary material for: Web-Based Intervention to Teach Developmentally Supportive Care to Parents of Preterm Infants: Feasibility and Acceptability Study
Source: JMIR Res Protoc. 2017 Nov 30;6(11):e236. doi: 10.2196/resprot.8289 (PMC5730819; doi:10.2196/resprot.8289)
Supplement: Multimedia Appendix 1 [file resprot_v6i11e236_app1.pdf]

Multimedia appendix 2. User Satisfaction Questionnaire (n=45)

	Strongly disagree	Disagree	Agree	Strongly agree
The objectives of the tutorials were clear	0	0	27 (60%)	18 (40%)
The tutorials were well-organized	0	0	27 (60%)	18 (40%)
The material was presented in an interesting manner	0	1 (2%)	23 (51%)	21 (47%)
There were sufficient examples and illustrations	0	3 (7%)	26 (58%)	16 (36%)
The concepts were clearly presented and easy to understand	0	1 (2%)	23 (51%)	21 (47%)
The video examples were helpful in illustrating the concepts	0	0	23 (53%)	20 (47%)
The page setup was helpful in illustrating the concepts	0	0	30 (67%)	15 (33%)
The photos were helpful in illustrating the concepts	0	0	26 (58%)	19 (42%)
The tutorials increased my knowledge about my child’s demands and needs	0	1 (2%)	20 (44%)	24 (53%)
I feel capable of administering these techniques with my child	0	0	22 (49%)	23 (51%)
The length of the tutorials were appropriate	0	6 (13%)	22 (49%)	17 (38%)
This technology was as effective as traditional teaching methods in helping me learn the material	1 (2%)	4 (9%)	25 (56%)	15 (33%)
I would recommend this course to other parents of premature newborns	0	0	19 (42%)	26 (58%)
I enjoyed taking the tutorials	0	0	18 (40%)	27 (60%)
Overall, I was satisfied with the tutorials	0	0	24 (53%)	21 (47%)
