# Supplementary figures and images for: Web-Based Intervention to Teach Developmentally Supportive Care to Parents of Preterm Infants: Feasibility and Acceptability Study
Source: JMIR Res Protoc. 2017 Nov 30;6(11):e236. doi: 10.2196/resprot.8289 (PMC5730819; doi:10.2196/resprot.8289)

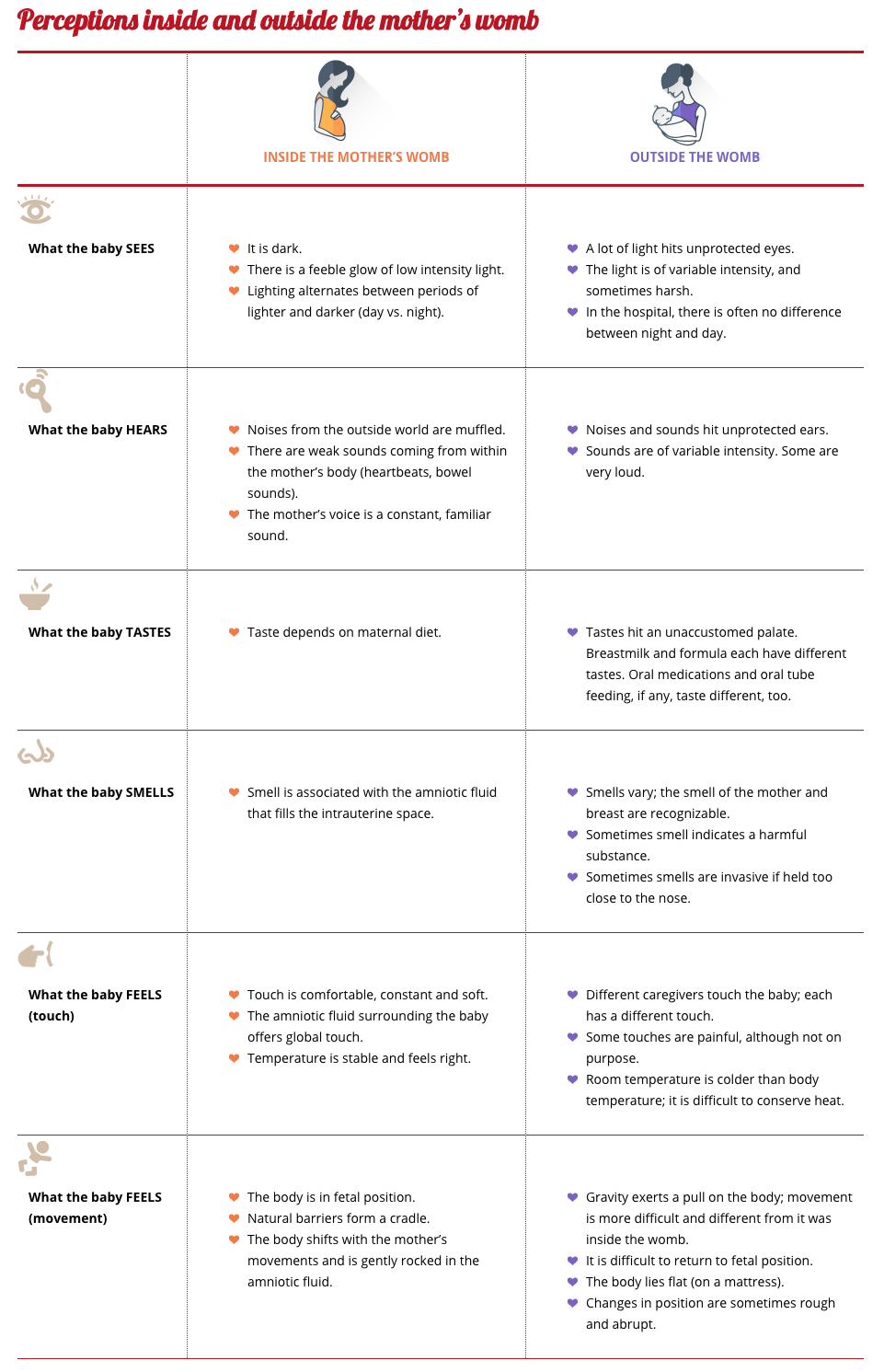

Supplement: Multimedia Appendix 2 [file resprot_v6i11e236_app2.png]

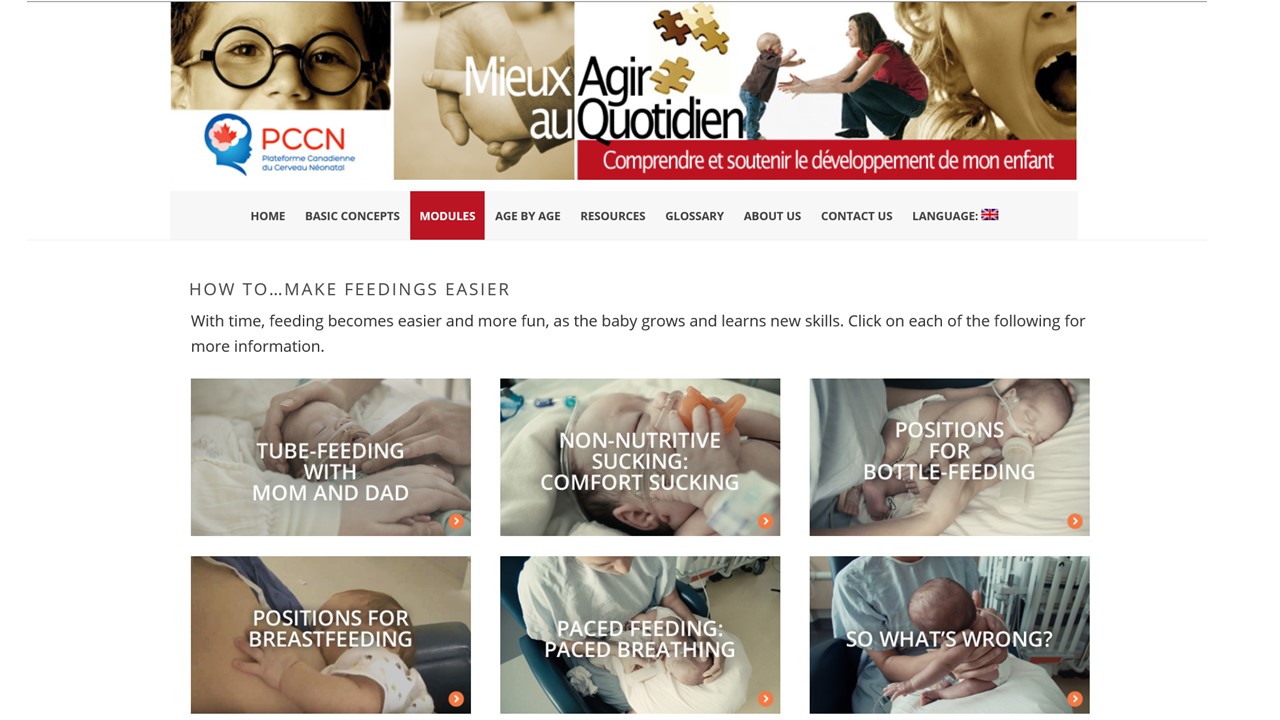

Supplement: Multimedia Appendix 3 [file resprot_v6i11e236_app3.jpg]
