# Supplementary material for: Web-Based Intervention to Teach Developmentally Supportive Care to Parents of Preterm Infants: Feasibility and Acceptability Study
Source: JMIR Res Protoc. 2017 Nov 30;6(11):e236. doi: 10.2196/resprot.8289 (PMC5730819; doi:10.2196/resprot.8289)
Supplement: Multimedia Appendix 4 [file resprot_v6i11e236_app4.pdf]

SickKids®
FOUNDATION

June 14, 2010

Dr. Thuy Mai Luu
Department of Pediatrics, Neonatal Follow-Up Program
University of Montreal
3175 Chemin Cote-Sainte-Catherine
Montreal, QC H3T 1C5

Dear Dr. Luu,

Re: Grant No. *NI11-006 A Multi-Modal Approach to a Family-Centered Educational Program of Developmental Care for Preterm Infants: Development and Implementation*

On behalf of SickKids Foundation and the Institute of Human Development, Child and Youth Health – CIHR, I am delighted to inform you that your application has been approved an operating grant in the amount of $296,124 over a three year period. A mentorship award of $5,000 annually has been approved for the duration of the grant and will be directed to the mentor through the mentor's institution. I will provide you with the payment details once available.

Please indicate to us in writing that you accept this grant, that you have not received funding from any other source for the project (other than that shown in your application, if any), and the project's expected start date. The project's starting date must be within six months of the approval date.

Please take note of the enclosed "Grant Recipient Instructions", which outline your responsibilities in accepting this grant, including reporting responsibilities and acknowledgement of funding on all publications, presentations and media releases. For your reference, the review comments are also enclosed for your consideration.

While the New Investigators grants are partnered awards between SickKids Foundation and the Institute of Human Development, Child and Youth Health, the program is administered by the Foundation. Any questions you have about the award should be directed to me by email at pam.gilliland@sickkidsfoundation.com or by phone at 416-813-6166 x2354. We wish you continued success with your research.

Yours sincerely,

Pam Gilliland
Grants Officer, National Grants

cc: Anne-Cécile Desfaits, Assistant Director, CIHR, Institute of Human Development, Child & Youth Health (IHDCYH)

Encls.

525 University Avenue
14th Floor
Toronto, ON M5G 2L3
Phone 416 813 6166
Fax 416 813 5024
Toll Free 800 661 1083
www.sickkidsfoundation.com

CANADA REVENUE AGENCY
CHARITABLE BUSINESS NUMBER:
108084419RR0001

SickKids
FOUNDATION

Grant Recipient Instructions
New Investigator Research Grants Program

1. Upon notification of your grant please inform SickKids Foundation, by email, that you accept the grant and that you have not received funding from any other source for the project (other than that shown in your application, if any); include the project start date and the estimated completion date of the approved project.

   The Foundation does require various approvals, as appropriate to the research, be in place prior to the project start. Please note that we send the first payment to the research accounting office prior to receiving theses approvals. Grantees are responsible for making sure that their Research Ethics Approval (REB) and/or Animal Care Approval are up to date, and Health Canada approval, if required, is in place. **We do require that the sponsoring organization holds the payment for your project and releases funds once approvals are in place**. Please send a copy of approvals to the Foundation within one year of approval, or all payments made must be returned to the Foundation.

   Ownership and commercialization of any intellectual property developed during the research supported by this grant will be determined in accordance with the Intellectual Property policy of the Recipient Institution. For greater certainty, SickKids Foundation makes no claim to any intellectual property rights developed during the course of the study.

2. Any requests to change the funded project must be sent by email to the Foundation for approval. Include a brief yet thorough description of the factors necessitating the change. These changes include the following:

   2.1 Changes to the approved experimental protocol
   2.2 Changes in personnel, including Principal Investigators, Co-Investigators, Technicians and Laboratory Assistants
   2.3 Re-allocation of funds within the approved budget
   2.4 Extensions of the proposed timelines

3. Principal Investigators involved in projects that extend beyond one year must submit a progress report and financial statement (produced by the sponsoring institution's finance department) prior to the release of year two and year three funding. A sum of $10,000 of the third payment will be held back until receipt of the final report and financial statement.

3.1 Progress reports will be reviewed by members of the Grant Review Committee, and if approved, payment will be released.

3.2 Progress reports need not be lengthy, but should provide sufficient information to evaluate the progress based on the description of the project framework provided by the applicant in the original proposal.

4. All Principal Investigators are required to submit a final report and final financial statement (produced by the sponsoring institution's accounting department) upon completion of their investigations. This provision applies to all projects, whether two or three years in duration.

4.1 A satisfactory final report will consist of brief answers to the questions posed in the reporting guidelines, which will be sent out with the first payment.

4.2 Grant funds not expended for the purposes intended in the approved application should be refunded to the Foundation promptly.

4.3 Upon receipt of the final report, the Foundation will release the final payment of $10,000 on the grant.

5. The mentorship award of $5,000 annually is offered in recognition of the importance for New Investigators to receive the support and guidance that will yield the best possible research. The mentorship award will be directed to the mentor through the mentor's institution and may be used at the discretion of the mentor in support of the mentor's child health research activities. Although there is no time limit on spending the award, the mentorship prize payments will be subject to progress reporting on an annual basis for the duration of the grant.

6. Credit should be given in publications, presentations or communication activities including media, symposia, research reports and promotional events arising from work funded by this grant. Please note that support was given by "SickKids Foundation and Institute of Human Development Child and Youth Health - CIHR"; the grant title should be quoted. Please send an electronic copy of any publications arising from this grant to SickKids Foundation. Media releases should also note that SickKids Foundation, apart from government, is the largest funder in child health in the country. Please share media releases with the Foundation.

7. Researchers are encouraged to disseminate research project results to different users of knowledge, including the public, health care practitioners, the media, researchers, policy makers and others, using language, formats and media appropriate to each user group. Grant holders are encouraged to notify SickKids Foundation of any dissemination activities that they undertake including any symposia, conferences and any other promotional events. Similarly, the Foundation and IHDCYH- CIHR agree to notify the researcher about any dissemination activities that they undertake.

8. We ask that you refer to the Foundation grant number and grant title when communicating with the Foundation regarding a project.

9. If you have any further questions, or if you require clarification on any of these matters please contact the Foundation office at national.grants@sickkidsfoundation.com.

National Grants
SickKids Foundation

Applicant's Statement of Problem:
The statement of the problem could be clarified. Exactly what is the problem the authors wish to address in this study? It needs to be clarified and simplified to help the reader make the link between the interventions proposed and the effects wished for. For example, the authors propose to examine the effect of their intervention on parent-infant relationship but the link to the stated problem is absent.

The authors propose a family-centered educational program but do not clarify how the hospital or the home setting will be adapted to incorporate such an approach. Family-centered care is more than giving parents an education program or a list of activities to do at home, it means that the family is part of the decision-making process, is viewed as expert on the child, influences the type of care the child receives, and that services are individualized to the needs of each family, etc… Is the term "family-centered" appropriate for this study? The authors are proposing an educational program for parents, not the integration of the family into a plan of care which would be closer to the true meaning of family-centered care. It is confusing. The authors on p.5 of the proposal, later change their wording to "family-focused". There is a difference between those two appoaches; which one is appropriate for this study? Be consistent.

The use of the word "developmental care (DC)" is also confusing and needs to be clarified if used. Developmental care as it has been traditionally defined implies highly individualized nursing and medical care to an infant in the NICU with the inclusion of the family's needs as part of the considerations given in the determination of care. I am unclear as to how this study will provide developmental care as is traditionally known in neonatology. Maybe the authors need to use terms such "developmentally supportive" rather than DC. Part of the study seems to be in the NICU starting at 34 weeks, but it is unclear as to what will be done other than flexed positioning, interpretation of stress behaviors. Oral feeding support is also mentioned. Since the study continues after NICU discharge (as stated in p.5), the use of the term developmental care is questioned (and confusing) as it has never been used for that setting before. Do the authors rather mean "developmentally supportive" interventions/program?

Applicant's Knowledge of Literature:
The authors demonstrate good and current knowledge on the developmental outcome of prematurity and the effectiveness of intervention strategies on parental and child outcomes. The literature on family-centered care and developmental care is covered but the authors' interpretation of this work (and usage of the terms) for their study is not consistent with the literature.

Significance of Problem:
The problem is significant and deserving of funding of well designed research proposals.

Objectives of Project: *To develop, validate and test the feasibility of a family-centered educational program of developmental care for preterm infants (develop content; validate content; document effects).*

Ambitious project. It is difficult to judge the quality of this project/objectives as there is little information in the proposal on the content (topics) of the proposed intervention program.

Methodology/Research Design:
Maternal education and socio-economic status play a critical role in the developmental outcome of all children. Will the authors control for daycare/no daycare and quality of daycare post-discharge from the

NICU? The authors need to address how they will control for these variables in this study. Since the authors propose follow-up to age 24 months, these need to be controlled (although at other times in the proposal, the authors mention follow-up to 12 months).

Many questions regarding the "content" of the program as described on p.7. The authors offer very little specific details. For example: oral feeding support for one year; anticipatory guidance of preterm infant development and activities for 2 years! By whom, how often, and what? How will oral feeding support be individualized to each infant? I see no other way of delivering this support, same for anticipatory guidance.

Some subjects will receive the instructions via the web, others through handouts and videotapes. Do we know if these are equally effective education delivery methods? Are handouts also provided to the web-based families? How often will the person-to-person learning-teaching sessions be given?

The 2-phase design is acceptable and appropriate for this type of research (with control in the first phase). But is it realistic to propose the recruitment of 50 parent-infant dyads in a 5-month period? Twice? How will these families receive the kind of individualized attention critical to the success of this type of sensitive work given the large number recruited in such a short time period?

Since there are very few details as to the content of the educational program, I cannot comment on the appropriateness of the proposed intervention model. I am confused as to when the study would begin (when implemented). It is mentioned that the dyads would be recruited at 32 weeks but later it is mentioned the study would begin at discharge. Could the authors provide more clarification on what is intended to be done at different times, the topics covered, the modalities used?

The authors propose many outcome measures that are self-report or questionnaires filled by the parent; the level of education needed to complete those needs to be reported. Who is the parent- always the mother? Is the father considered as a parent only if primary caregiver?

A major concern is whether the examiners will be blind to group status or to study purpose. This has to be resolved in some manner that will provide integrity to the study's results.

Budget Suitability:
Should families be compensated for their participation? Will transportation be provided?

Clarity of Proposal:
Based on my comments on the methods section above, the proposal lacks in clarity.

Innovation:
Too much information is missing on the proposed educational program to determine its level of innovation.

Knowledge Translation Plan is appropriate to nature of research: 1-improve knowledge on preterm child development and DC and 2- explore their perceptions on implementation of such programs.
2- hour course (pre and post- quiz and 6 months later) and focus groups (1 hour discussions)

A 2 hour workshop hardly seems enough to reach the goals set by the authors. On-going training, mentoring and reflective practice would be more adequate for such delicate work with complex infants and families.

Overall project weaknesses:<br>Confusing use of terminology (family-centered care and developmental care).<br>Choice of outcome measures not always obvious based on purpose, goals and objectives of project.<br>Weak methodology.
Overall project strengths:<br>The authors propose to do a NICU to home program which is missing in the literature and intervention programs. Very important strength of this proposal.

Applicant's Statement of Problem:<br>A concise statement of important issues regarding the care and developmental support of preterm infants in the NICU and on followup once they are discharged home. The strong conclusion summarizes these issues, and the rationale as well as the hypotheses of this research project.
Applicant's Knowledge of Literature:<br>Excellent review of the relevant literature, the rationale for Developmental Care in the NICU, a succinct summary of early interventions after NICU discharge, and the benefit of programs that involve parents.
Significance of Problem:<br>The developmental support of preterm infants is extremely important. The focus for too long has been on their survival. Their neurodevelopmental problems have been well documented. It is now time to focus on ways to better support their development and to support their parents.
Objectives of Project:<br>It makes sense to involve parents in providing developmental support, because it is very clear that infants thrive in an enriched environment. Multiple teaching modalities is very innovative, especially those using web-based technologies.
Methodology/Research Design:<br>Thoughtful and well designed. At the end of this project, they will have developed a family-centered educational program of Developmental Care for preterm infants that has been validated and piloted in a clinical trial.
Budget Suitability:<br>Seems very reasonable.
Clarity of Proposal:<br>Well stated objectives, methodology and plan integrating what they develop into their clinical program.
Innovation:<br>Using web-based technology to reach out to parents and teach them about their infant's development is very innovative.
Knowledge Translation Plan is appropriate to nature of research:<br>They have an excellent plan to involve nurses in developing teaching modules and reaching parents,and to evaluate barriers to implementing their program for parents.
Overall project weaknesses:<br>The only weakness is one we all encounter when testing a developmental intervention program: it is impossible to do a randomized controlled trial, so we are left with using historical controls. I know of no way to avoid this.
Overall project strengths:<br>This project addresses a very important issue: how can we best support the development of preterm infants? The plan to involve parents gets to the heart of the issue, and their approach of using different modalities shows great sensitivity and insight into the problems parents of NICU infants and individual variability in how adults learn.

Applicant's Statement of Problem:<br><br>Statement of the problem is clear and concise.
Applicant's Knowledge of Literature:<br><br>Outstanding knowledge of literature.
Significance of Problem:<br><br>The problem is very timely as many provinces are examining early developmental interventions as "sustainable cost avoidance".
Objectives of Project:<br><br>In essence, this is a pilot project to determine the feasibility of a randomized trial, excellent scientific methods followed.
Methodology/Research Design:<br><br>While this is a non-random design, it is perfectly appropriate for a feasibility trial.
Budget Suitability:<br><br>I was initially concerned about salaries (e.g. OT at $22/hr), I was assured that this is a standard in Quebec (very low by national standards).
Clarity of Proposal:<br><br>The proposal plans to develop over the three years and the investigators have provided a clear outline of how they plan to get there.
Innovation:<br><br>There are examples of early interventions examining improved outcomes, although very few start in NICU and follow through after discharge.
Knowledge Translation Plan is appropriate to nature of research:<br><br>Generally pretty good, however, the community beyond Sainte-Justine would like to hear about the outcomes. Thus, presentation at major meetings and peer reviewed publications would be necessary.
Overall project weaknesses:<br><br>- minor point: it is the Bayley Scales of Infant and Toddler Development (BSITD), they used the name of the old version.<br><br>- short term outcome only (at 12 months)
Overall project strengths:<br><br>- very timely topic.<br><br>- these interventions may improve development of the children potentially improving long-term outcome and could lead to "sustainable cost avoidance". This would require a larger trial to show.

Hospital for Sick Children Foundation/CIHR IHDCYH
New Investigator Research Grant Review

Grant Number: **NI11-006**
Title: **A Multi-Modal Approach to a Family-Centered Educational Program of Developmental Care for Preterm Infants**

Principal Investigator: **Thuy Mai Luu**

1. Project Summary

This is a prospective historical cohort study whose target population is preterm infants under the age of 29 weeks gestation who survive to a gestational age of 32 weeks. This study proposes the development, validation, and implementation of a multi-modal family-focused educational program of developmental care. The rationale is that increasing parental knowledge of preterm child development and increasing parental awareness of parenting skills that support developmental beneficial infant practices will improve the outcome in preterm infants who are at high risk for adverse neurodevelopmental sequelae. The project is based at Hopital Sainte Justine with subject recruitment from the neonatal intensive care unit of that hospital. Approximately 50 children will be included in each group. One group is a historical control and the second group is a prospectively enrolled cohort. These respective cohorts are to be enrolled over sequential and overlapping six month intervals and represent roughly 80 to 85% of the eligible population encountered in Hopital Sainte Justine's NICU. The primary outcome measure is infant cognitive development at one year's corrected age with a variety of secondary outcome measures also selected primarily addressing parent infant interactions, infant stress and parental competency. The principal investigator is a physician who graduated from McGill University in 2001 and then completed a 5 year residency in Pediatrics at Hopital Sainte Justine. Subsequently she completed a one year fellowship in neonatal follow-up at Brown University in Providence, Rhode Island. She has also completed an MSc in Epidemiology at McGill University. She presently holds a small amount of operating funds as a start-up grant from Hopital Sainte Justine. Her academic appointment began as a clinical lecturer in November 2008. Her publication record is very limited, with three publications one of which is a case report. The remaining two publications, are in a high impact factor journal and directly addresses the issue of neurodevelopmental outcome in premature infant survivors

2. In-depth Comments and Review

This is a prospective historical cohort study. Its target population is one that is at considerable risk for adverse lifelong neurodevelopmental morbidity. The proposal itself is well-written demonstrating a good grasp and summary of the relevant literature. While the principal investigator has limited research experience and productivity, a multi-disciplinary collaborative team has been assembled in support of this particular research effort. The study itself is highly appropriate and relevant to child health. Indeed preterm births are now approaching approximately 10% of all births in our population and such

Grant Number: **NI11-006**
Title: **A Multi-Modal Approach to a Family-Centered Educational Program of Developmental Care for Preterm Infants**

Principal Investigator: **Thuy Mai Luu**

children have a 50% risk of a lifelong neurodevelopmental disability. The project is based on the supposition that improved parental knowledge of preterm development and an awareness of appropriate parenting skills that support developmentally beneficial practices will improve infant outcome. The primary outcome measure is cognitive development as measured by standardized testing (Bayley's) at one year of age. Additional outcome measures are employed at a secondary level to assess infant development, infant stress, parent-infant interaction, parental competence, parental stress and parental emotional outcomes. Roughly 50 children will be included in each arm of the study, representing subject recruitment of approximately 80% of eligible infants in Hopital Sainte Justine NICU. A power calculation is provided to reveal that these are sufficient numbers to generate the expected estimated effect of this educational intervention. The educational program itself is not yet formulated nor validated nor indicated as being feasible. Indeed these efforts will comprise the first part of this project. It is stated that this educational program will account for different adult learning styles and will utilize new technologies such as the Internet and web-based modules.

The project is an ambitious one and is hampered by the lack of an as-yet developed educational program which would be the independent variable of interest. As such there is no pilot data and there is no demonstration of the feasibility of this educational program. It is stated that the results of this project will be preliminary data that will provide the basis for a subsequent pilot clinical trial and hopefully an eventual multi-centre randomized controlled trial (RCT). The principal investigator does not demonstrate a strong track record of research achievement, though there are highly supportive letters in the file from the Chair of Pediatrics of Universite de Montreal and the Director of the Applied Research Center at Hopital Sainte Justine's Research Institute. The lack of possible blinding in terms of the application of the outcome measures is also somewhat problematic. There is an obvious bias in the research design to detecting a positive effect as a result of any intervention implemented.

3. <u>External Reviews</u>

All the external reviews highlight that the proposal is well-written with an excellent review of the literature. The reviewers all highlight that the project addresses an important problem in child health. There are comments regarding the imprecise use of terminology and the lack of precise details regarding the educational program prior to its application. Scientific merit is in the "very good" range predominantly and the project utility as a feasibility study as preliminary to larger randomized controlled trials is highlighted.

Grant Number: **NI11-006**
Title: **A Multi-Modal Approach to a Family-Centered Educational Program of Developmental Care for Preterm Infants**

Principal Investigator: **Thuy Mai Luu**

4. Summary of own comments

This is a well-written ambitious proposal by a Principal Investigator early in her career with limited, indeed modest, research accomplishments to date. The project addresses an important problem in child health and is innovative. It is lacking in the formulation and details regarding the educational program that is the target intervention and it is also entirely lacking in pilot and feasibility data.

5. Budget

The budget is roughly $300,000 over a three year interval. Funds set aside for the involvement of an occupational therapist and a physiotherapist for application of some of the standardized outcome measures appears to be an under-estimate. Otherwise the budget provided appears to this reviewer to be appropriate.
